# Effects of habitat gradient and agro-climatic variation on selected soil physical and chemical properties in the Bale Mountains national park, south-eastern Ethiopia

**DOI:** 10.1186/s12862-022-02032-7

**Published:** 2022-06-20

**Authors:** Annissa Muhammed Ahmedin, Eyasu Elias

**Affiliations:** 1 Centre for Environmental Science, College of Natural and Computational Science, Addis Ababa University, 1176 Addis Ababa, Ethiopia; 2 Department of Natural Resource Management, College of Agriculture and Environmental Science, Arsi University, 193 Assela, Ethiopia

**Keywords:** Agro-climatic variation, Habitat gradients, Landscape change

## Abstract

**Background:**

Increasing evidence suggests that anthropogenic effects are responsible for drastic changes in landscape patterns and ecosystem services. This study aims to assess the effects of landscape change and agro-climatic variation on selected soil physical and chemical properties in the Bale Mountains national park. A combination of stratified and systematic sampling techniques was employed to draw representative soil samples. A total of 72 soil samples (3 agro-climatic zones × 3 land cover types × 2 habitat gradients × 4 replications = 72) at a depth of 0–20 cm were collected for the soil physical and chemical property analysis. A two-way analysis of variance was conducted to determine the level of variation in soil parameters. Tukey’s honest significance difference (HSD) test was used to compare treatment means at a 0.05 level of significance.

**Results:**

The results suggest that soil parameters differed significantly (*p* < 0.05) among agro-climatic zones, land cover, and habitat gradients. The soil pH, SOC, TN, AP, CEC and clay content were significantly higher in the lower altitude, natural vegetation and interior habitat, whereas the soil sand and silt content as well as the soil bulk density were significantly higher in the farmland and edge habitat.

**Conclusions:**

Conservation and restoration priority should be given to those vegetation types and ecosystems that are highly affected by human interferences such as the grassland in the middle altitude, ericaceous land in the higher altitude, and moist forest in the lower altitudes.

## Introduction

Habitat loss and fragmentation are among the most serious challenges to the terrestrial ecosystem [1]. Growing evidence implies that human-caused changes are responsible for radical changes in landscape pattern and ecological services [2]. The effect of anthropogenic activities on the natural ecosystem becomes accelerated mainly due to the growth of human population and urban sprawl. Moreover, extreme human disturbances had a substantial impact on landscape structure and function on a worldwide scale [3, 4]. Fluxes in soil physicochemical properties, such as soil temperature and nutrient contents, are triggered by habitat fragmentation, resulting in changes in the structure and metabolic performance of microbial communities, with implications for soil nutrient cycling [5]. Conversely, different types of land use have varying degrees of impact on soil quality. At the same time, different landscapes contain various types of land uses along elevation gradient with different plant composition and ecosystem structure [6]. The more the land is occupied by diverse range of species, the more the soil will become fertile [7]. Even within the same land use type but different floristic composition may generate different soil properties [8]. Elevation gradient and agro-climatic zonation may have profound effect on soil properties as well. The main reason for the variation of soil quality along elevation gradient and floristic composition pertains to control of soil erosion and runoff and nutrient losses [9]. These problems may get aggravated as the landscape gets devoid of vegetation cover induced by anthropogenic land use changes. Since soil properties are considerably affected by net primary productivity, it is predictable that the potential effects of habitat fragmentation over biomass availability may impact nutrient cycling in the soil (e.g., C, N and P cycling) [10]. There are very few studies that have evaluated the effects of landscape change and agro-climatic variation on the soil system functioning.

The Bale Mountains national park (BMNP) is characterized by high levels of species richness and endemism [11]. The park was established in 1970 after the British naturalist Dr. Leslie Brown’s recommendation during his two visits in 1963 and 1965 to assess the status of Mountain nyala. The vegetation of the region is distinct in character, highly specialized and exposed to a wide range of environmental conditions, with steep ecological gradients that are dependent on topographic aspect, slope, climate and soil conditions [12]. In spite of its huge potential and ecological importance, habitat degradation takes place at an alarming rate in different agro-climatic zones and poses a severe threat to the ecosystem [13]. The BMNP's landscape structure is gradually fragmenting, and human activities such as settlement expansion and subsistence agriculture have an impact on plant diversity and composition [14]. Many of the studies in the BMNP focuses on the extent of land use/land cover change and its effect on plant diversity and structure. However, this research attempt to answer the research questions including; What are the potential impact of habitat gradient and agro-climatic variation on the selected soil physical and chemical properties in the BMNP? What are the causes of it? and What measures to be taken to reverse this change?

## Materials and methods

### Study area description

**Fig. 1:**
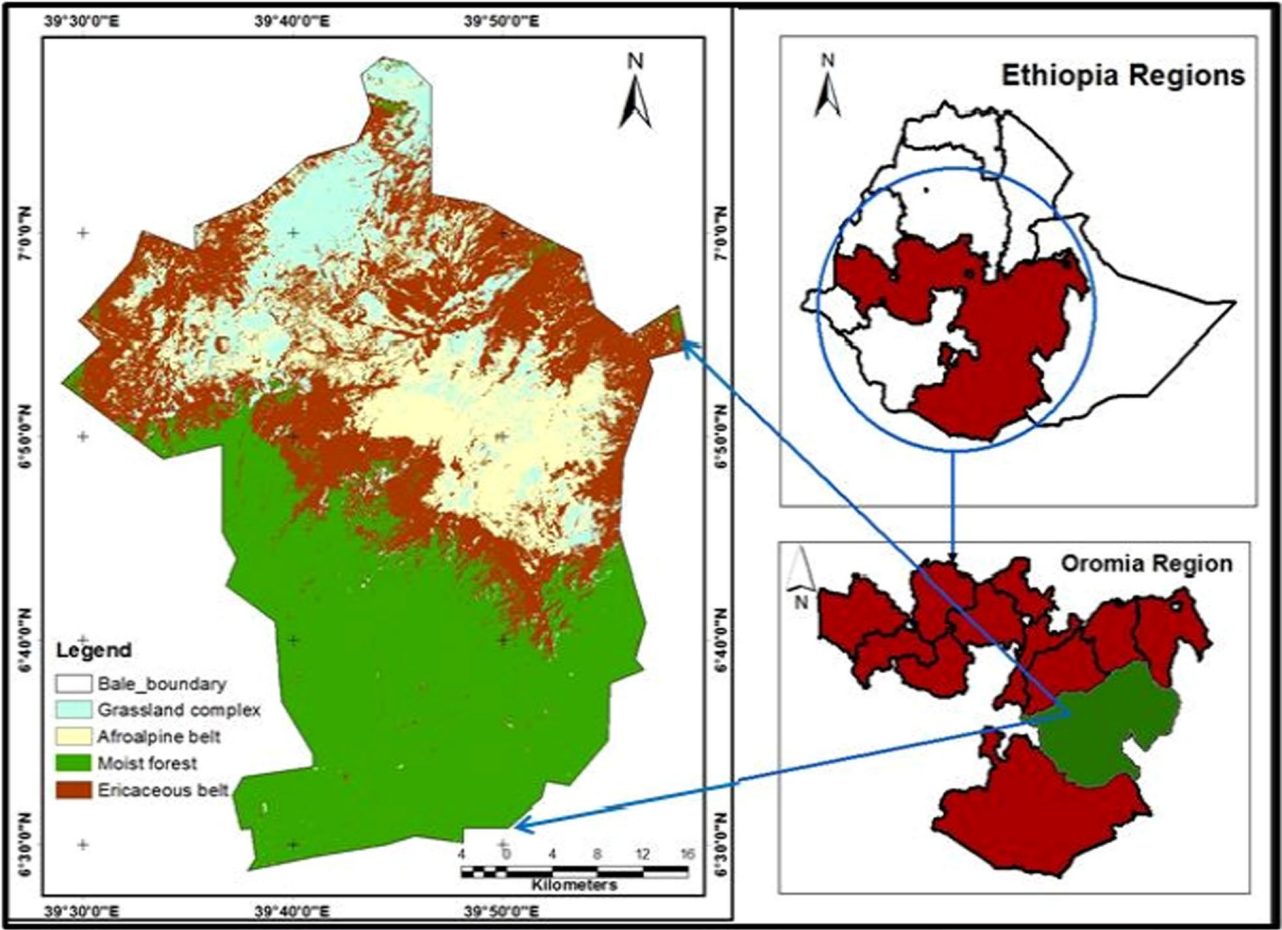
The location map of the study area

The Bale Mountains national park is located 400 km southeast of Addis Ababa in the Bale zone of Oromia National Regional State, Ethiopia. It encompasses 2178 km^2^ and is situated within the geographic bounds of 6°29ʹ–7°10ʹ N latitude and 39°28ʹ–39°57ʹ E longitude. Geologically, the Bale massif consists of Tertiary (Oligocene) lavas, overlying the Mesozoic marine sediments by underlying the Precambrian rocks after the Eocene uplifting of the Ethiopian highlands. The major soil types within the BMNP are Chromic Luvisols (32.54%) covering extensive areas in the gently sloping foothills below the escarpment. Pellic Vertisols (21.84%) cover the second largest area, mainly in the low-lying lands that have seasonal drainage deficiencies. In the gently sloping area, eutric nitisols cover an area of 16.85%. The higher elevation plains of the BMNP are covered by well-drained Eutric Cambisols and Orthic Luvisols, accounting for 16.62% of the area. Leptosols in the steep slopes and mountain ranges cover 7.69% of the area. On the escarpment, 5% of the area is deep, well-drained Orthic Luvisols and Dystric Histosols (Fig. 1).

### Sampling design

**Fig. 2:**
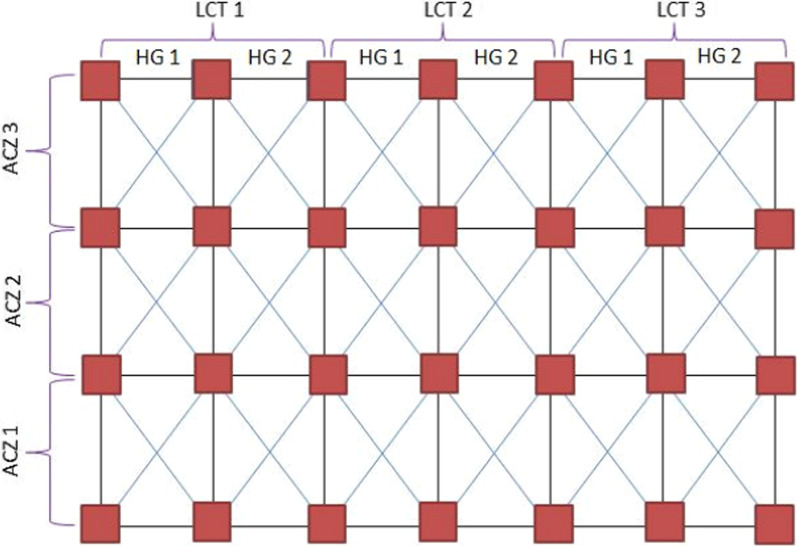
Soil sampling design. *ACZ*  agro-climatic zone, *LT*  land cover type, *HG*  habitat gradient

A combination of stratified and systematic sampling methods was employed to draw representative soil samples from different altitudinal zones, land use/land cover and habitat gradients following Bonham [15]. The study area was first stratified into three agro-climatic zones (ACZ) based on the agro-climatic classification of Ethiopia [16, 17] as Tepid sub-moist mid highland (1600–3000 m) which is designated as ACZ 1; the Cool moist mid highlands (3000–3400 m) that is designated as ACZ 2; and the cold humid afro-alpine zone (3800–4200 m) which denoted as ACZ 3. Then three land use/land cover types, such as forest land herbaceous land and farmlands were identified at each agro-climatic zones. Subsequently, each land use/land cover type was divided into two habitat gradients as edge and interior habitat. Soil samples were collected at four replications from each habitat gradient. Accordingly, a total of 72 soil samples (3 ACZ × 3 land cover types × 2 habitat gradients × 4 replications = 72) were collected. Equal number of soil samples was considered for the comparison of soil properties in the edge and interior habitats (Fig. 2).

### Soil sample preparation

Soil samples were drawn at a depth of 30 cm with a soil auger of 10 cm in diameter for soil physical and chemical property assessment. The soil samples were drawn from five points and homogenized manually to obtain a composite sample after the roots and coarse plant debris were removed and stored in white polythene bags. The initial weight of the soil samples was measured in-situ immediately after collection and its moisture content was determined gravimetrically by air drying the soil samples in a ventilated room until a constant weight was obtained. Simultaneously, the bulk density of the soil samples was computed. Prior to soil physical and chemical property analyses, the soil samples were crushed to pass through a 2 mm mesh sieve, then the samples were sent to Kulumsa agricultural research center for soil physical and chemical property analysis.

### Soil property analysis

Soil samples collected from the field were analyzed for some selected soil physical and chemical properties following the standard procedures. The soil texture (particle size fractions such as sand, silt, and clay expressed as % weight) was determined through the Boycous hydrometer method after dispersion in a mixer with sodium hexametaphosphate [18]. Bulk density (BD) was determined using a volumetric cylinder and calculated by dividing the oven dry mass at 105 °C by the volume of the core [19]. The soil pH was measured by combined glass electrodes in a 1:2.5 soil to water suspension as described by van Reeuwijk [20]. Soil organic carbon (SOC) was determined according to the Walkley and Black method [21] using a LECO-1000 CHN analyzer. Total nitrogen (TN) was measured following the macro-Kjeldahl method [22]. Available phosphorous (AP) was estimated using the Olsen method [23]. Cation exchange capacity (CEC) was analyzed titrimetrically through the distillation of ammonium displaced by sodium [24].

### Statistical data analysis

The statistical analysis of soil data was made using R statistical software version 3.5.2 for windows 8 [25]. Mean comparison among land cover types, altitudinal zones and habitat gradients were made using two-way analysis of variance (two-way ANOVA) following the generalized linear model (GLM) procedure. Tukey’s honest significance difference (HSD) test was used for mean separation when the analysis of variance showed statistically significant differences. In all statistical testing, significant differences at *p* < 0.05 levels were reported as statistically significant.

## Results

### Soil physical and chemical properties across the land cover types

The soil textural fractions were varied significantly (*p* < 0.05) among the land cover types. Overall, the soil texture falls under sandy loam class under the grassland, afro-alpine, ericaceous land and woodland while sandy clay loam texture dominates under coffee forest, moist forest and farm land (Table 1). The BD of the soil is generally in the very low category (0.39—0.96 g/m^3^) suggesting that the soil is not compacted yet under all the land use types and vegetation covers. Even then, there are statistically significant difference among land use types in the mean BD with the highest (0.96 g/m^3^) under the moist forest and the lowest (0.39 g/m^3^) under the ericaceous land. The mean sand content of the woodland (58.63 ± 2.50) was significantly (*p* < 0.05) higher compared to the soil in the other land cover type except the soil under the ericaceous land (58.74 ± 2.63). The mean clay content under moist forest (31.48 ± 1.51) was significantly (*p* < 0.05) higher compared with the soil under the other land cover types (Table 1). Moreover, the mean silt content of the ericaceous land (35.30 ± 2.61) was significantly (*p* < 0.05) higher compared with the soil under the other land cover types. Conversely, the mean Si/Cl ratio under ericaceous land (5.92 ± 0.08) was significantly (*p* < 0.05) higher compared with the soil under the other land cover types. The texture of the soil was dominated by sandy loam in most land use types. The mean value of soil texture fractions across land cover types were sand > silt > clay except in the moist and coffee forest, i.e., sand > clay > silt.

**Table 1:** The mean (± SE) values of selected soil physical properties as affected by land use types

Land cover type	Sand (%)	Silt (%)	Clay (%)	Si/Cl	Texture class	BD (g/cm^3^)
Grassland	55.59 ± 1.67^b^	27.22 ± 1.21^b^	17.19 ± 1.12^cd^	1.63 ± 0.13^b^	SL	0.65 ± 0.05^b^
Afro-alpine	53.35 ± 2.13^b^	27.31 ± 1.29^b^	19.34 ± 1.05^c^	1.43 ± 0.07^b^	SL	0.48 ± 0.04^bc^
Ericaceous land	58.74 ± 1.02^a^	35.30 ± 1.15^a^	5.96 ± 1.01^e^	7.55 ± 1.39^a^	SL	0.39 ± 0.04^c^
Woodland	58.63 ± 1.11^a^	27.96 ± 1.85^b^	13.41 ± 1.07^d^	2.25 ± 0.30^b^	SL	0.57 ± 0.07^b^
Coffee forest	53.45 ± 1.16^b^	21.69 ± 1.26^c^	24.86 ± 1.42^b^	0.90 ± 0.09^c^	SCL	0.88 ± 0.11^ab^
Moist forest	53.24 ± 2.37^b^	15.28 ± 1.03^d^	31.48 ± 1.51^a^	0.49 ± 0.02^d^	SCL	0.96 ± 0.14^a^
Farmland	53.08 ± 1.18^b^	25.82 ± 0.73^b^	21.11 ± 1.08^c^	1.30 ± 0.08^b^	SCL	0.72 ± 0.08^ab^
Mean	54.69 ± 0.64	25.80 ± 0.73	19.51 ± 0.93	2.02 ± 0.28	–	0.68 ± 0.04
*F*	5.10	71.16	51.73	43.88	–	6.51
Sig	*	***	***	***	–	**

Means in each column with the same superscript letter are not significantly different at *P* ≤ 0*.*05; *SL*  sandy loam, *SCL* sandy clay loam, *BD* bulk density; ^∗∗∗^*p* < 0*.*001, ^∗∗^*p* < 0*.*01 and ^∗^*p* < 0*.*05

Most of the soil chemical properties investigated were significantly (*p* < 0.05) affected by land use types (Table 2). The soil pH ranges from 5.14 to 5.97 that can be rated as strongly acid to moderately acid based on the ratings proposed by Landon [26]. The strongly acidic soils were found under farmland (5.14 ± 0.02), grassland (5.29 ± 0.09) and afro-alpine (5.33 ± 0.16) land use types while all other land cover types have moderately acidic soil reaction.

**Table 2:** The mean (± SE) values of selected soil chemical properties as affected by land use types

Land cover type	pH	SOC (%)	TN (%)	C/N ratio	AP (mg/kg)	CEC (cmol/kg)
Grassland	5.29 ± 0.09^c^	6.21 ± 0.46^a^	0.51 ± 0.04^b^	12.27 ± 0.45^ab^	5.05 ± 0.23^b^	15.03 ± 1.65^b^
Afro-alpine	5.33 ± 0.16^c^	3.89 ± 0.13^b^	0.33 ± 0.04^c^	12.70 ± 1.36^ab^	3.39 ± 0.14^b^	16.41 ± 1.35^b^
Ericaceous land	5.97 ± 0.17^a^	7.40 ± 0.54^a^	1.08 ± 0.17^a^	7.43 ± 0.63^c^	17.69 ± 2.24^a^	31.63 ± 2.42^a^
Woodland	5.53 ± 0.18^bc^	7.23 ± 0.69^a^	0.81 ± 0.15^a^	10.05 ± 0.99^b^	18.96 ± 2.07^a^	25.95 ± 2.39^a^
Coffee forest	5.79 ± 0.35^ab^	6.96 ± 0.67^a^	0.48 ± 0.04^b^	14.50 ± 0.52^a^	14.14 ± 1.68^a^	15.71 ± 1.98^b^
Moist forest	5.81 ± 0.12^ab^	7.14 ± 0.52^a^	0.50 ± 0.09^b^	16.30 ± 1.91^a^	17.18 ± 1.98^a^	28.50 ± 2.21^a^
Farmland	5.14 ± 0.07^c^	3.75 ± 0.27^b^	0.43 ± 0.03^bc^	9.01 ± 0.58^b^	1.19 ± 0.12^b^	14.57 ± 1.02^b^
Mean	5.47 ± 0.06	5.54 ± 0.25	0.56 ± 0.04	11.14 ± 0.48	8.89 ± 0.98	19.66 ± 0.99
*F*	4.80	19.65	12.44	7.78	15.39	14.42
Sig	*	***	***	**	***	***

Means in each column with the same superscript letter are not significantly different at *p* ≤ 0*.*05; *SOC* organic carbon, *TN*  total nitrogen, *C/N*  carbon to nitrogen ratio, *AP*  available phosphorus, *CEC*  cation exchange capacity; ^∗∗∗^*p* < 0*.*001, ^∗∗^*p* < 0*.*01 and ^∗^*p* < 0*.*05

The content of SOC showed highly significant variability across different land use types with the highest (7.4%) under the ericaceous land and the lowest (3.75%) under the farmland. Generally, the SOC content of the study area is in the range of medium to high according to the ratings proposed by Landon [26]. The lowest SOC under the cultivated fields is expected because of complete removal of crop residues from fields and farm yard manure burning as household energy instead of recycling back to the soil. The TN content follows exactly the same pattern to the SOC with the highest (1.08%) under ericaceous land and the lowest 0.33 and 0.43 under Afro-alpine and farmland, respectively. Generally, the soil TN content is rated as medium to high according to Landon [26]. The C/N ratio in the coffee forest (14.50 ± 1.26) and moist forest (14.28 ± 1.08) was significantly (*p* < 0.05) higher compared to the soil in the other land cover types except the grass land (12.17 ± 0.65) and afro-alpine land (11.78 ± 0.31).

The AP content in the soil ranges from 1.19 mg/kg in the farmland to 18.96 mg/kg in the woodland ecosystem showing highly significant (*p* < 0.01) variation across land use types. Overall, the AP content of the soil is rated as low under farmland and afro-alpine land, medium under grassland and coffee forest and high under woodland, ericaceous land and moist forest. The AP content somehow reflects variations in the soil pH. AP is lowest in the land use types with lowest pH suggesting the problem of P-fixation in the strongly acidic reactions.

The CEC ranges from as low as 14.57 cmol/kg in the soils under farmland to as high as 31.63 cmol/kg in the soils under ericaceous land. Overall, the CEC of the soil is rated as medium under farmland, grassland, Afro-alpine forest and coffee forest but it is high under Ericaceous land, woodland and moist forest. This is a mirror image of the distribution of the SOC content of the soil. As would be expected, the CEC of the soils under ericaceous land, woodland and moist forest is high suggesting the contribution of the organic matter to the CEC in addition to the soil clay minerals.

### Effects of agro-climatic variation on soil physical and chemical properties

The influence of agro-climatic variation was significant (*p* < 0.05) in most of the soil physical and chemical properties (Table 3). Accordingly, the mean sand content in the agro-climatic zones of cool moist mid highlands (ACZ 2) (57.81 ± 2.29) and very cold humid afro-alpine zone (ACZ 3) (55.87 ± 2.68) was significantly higher compared to the soil in the tepid sub-moist mid highlands (ACZ 1) (53.23 ± 2.10). Conversely, no significant difference in the mean sand content was found between ACZ 2 and ACZ 3. The highest sand content in ACZ 2 might be due to its steep slope (30–60%) compared to the other two agro-climatic zones. Fine soil particles can easily be eroded by water in the higher slopes. The mean clay content between ACZ 1 (29.27 ± 2.59), ACZ 2 (14.67 ± 1.28) and ACZ 3 (12.80 ± 1.95) was significantly varied at *p* < 0.05. The highest clay content in ACZ 1 could be due to its lower slope position contributes for the retention of fine particles in the soil as a result of low level of soil erosion. In addition, the mean silt content among ACZ 1 (17.50 ± 1.54), ACZ 2 (27.51 ± 1.13) and ACZ 3 (31.56 ± 2.19) was significantly different at *p* < 0.05. The mean values of Si/Cl ratio followed exactly the same pattern to the silt contents of the soil in the agro-climatic zones. Conversely, the mean BD of the soil in the agro-climatic zones showed significant variation and followed similar pattern as the clay content with the highest in ACZ 1 (0.95 ± 0.06) and the lowest in ACZ 3 (0.43 ± 0.03).

**Table 3:** The mean (± SE) values of selected soil physical and chemical properties as affected by agro-climatic zones

Soil properties	Agro-climatic zones			Mean	*F*	Sig
	ACZ 1	ACZ 2	ACZ 3			
Sand (%)	53.23 ± 1.24^b^	57.80 ± 0.95^a^	55.64 ± 1.33^a^	55.56 ± 0.72	30.55	***
Silt (%)	17.50 ± 0.83^c^	27.53 ± 1.01^b^	31.56 ± 1.27^a^	25.53 ± 1.05	71.50	***
Clay (%)	29.27 ± 1.09^a^	14.67 ± 0.82^b^	12.80 ± 1.89^c^	18.91 ± 1.32	90.72	***
Si/Cl	0.62 ± 0.05^c^	2.01 ± 0.17^b^	4.49 ± 1.04^a^	2.37 ± 0.41	38.07	***
BD (g/cm^3^)	0.95 ± 0.09^a^	0.61 ± 0.04^b^	0.43 ± 0.03^c^	0.66 ± 0.04	45.41	***
pH (H_2_O)	5.81 ± 0.18^a^	5.44 ± 0.11^b^	5.32 ± 0.03^b^	5.52 ± 0.08	32.35	***
SOC (%)	7.02 ± 0.40^a^	6.73 ± 0.42^b^	6.19 ± 0.61^c^	6.65 ± 0.28	8.49	**
TN (%)	0.81 ± 0.15^a^	0.68 ± 0.09^a^	0.49 ± 0.15^b^	0.66 ± 0.06	7.74	**
C/N ratio	14.70 ± 2.75^a^	10.90 ± 0.63^b^	14.03 ± 1.52^a^	13.21 ± 1.07	31.91	**
AP (mg/kg)	18.79 ± 0.99^a^	15.13 ± 2.22^a^	5.54 ± 0.58^b^	13.15 ± 1.15	50.50	***
CEC (cmol/kg)	25.27 ± 2.20^a^	22.10 ± 2.18^ab^	20.49 ± 1.99^b^	22.62 ± 1.23	2.63	ns

Means in each column with the same superscript letter are not significantly different at *p* ≤ 0*.*05; *ACZ* agro-climatic and vegetation zone, *BD*  bulk density, *SOC* organic carbon, *TN* total nitrogen, *C/N* carbon to nitrogen ratio, *AP* available phosphorus, *CEC* cation exchange capacity, *ns* non-significant; ^∗∗∗^*p* < 0*.*001 and ^∗∗^*p* < 0*.*01

The soil pH across the agro-climatic zones was varied significantly (*p* < 0.05). Accordingly, the mean value of soil pH in the agro-climatic and vegetation zone of ACZ 1 (5.81 ± 0.15) was significantly higher compared to the soil pH in the ACZ 3 (5.32 ± 0.14) and ACZ 2 (5.44 ± 0.13). Conversely, no significant difference in the mean soil pH was found between ACZ 2 and ACZ 3. The soil pH can be rated as strongly acid and moderately acid based on the ratings proposed by Landon [26]. The strongly acidic soils were found in ACZ 3 and ACZ 2 while moderately acidic soil reaction was found in ACZ 1.

The SOC content showed significant variation (*p* < 0.05) across the agro-climatic zones with the highest (7.02%) under ACZ 1 and the lowest (6.19%) under ACZ 3. Generally, the SOC content can be rated as medium in all agro-climatic zones according to the ratings proposed by Landon [26]. The highest SOC content in the ACZ 1 could be due to the gentle slope (< 5%) and the relatively higher temperature in the area. The lowest SOC content under ACZ 3 could be due to the very low temperature in the area. The SOC in the ACZ 2 was the second least and that could be due to the steep slope (30–60%) of the area. Higher slope contributes for easy removal of fine particles from the soil while lower slope contributes for the retention of fine particles in the soil. In addition, higher temperature facilitates the decomposition rate of soil organic matter while lower temperature retards the decomposition rate of organic matter in the soil. The TN content of the soil across the agro-climatic zones follows exactly the same pattern to the SOC content. Accordingly, the mean TN content under ACZ 1 (0.81 ± 0.08) and ACZ 2 (0.68 ± 0.04) was significantly (*p* < 0.05) higher compared to the soil under ACZ 3 (0.49 ± 0.02). However, there was no significant difference in the mean TN content of the soil between ACZ 1 and ACZ 2. Generally, the soil TN content across the agro-climatic zones is rated as medium in the ACZ 3 and high in the ACZ 1 and ACZ 2 according to Landon [26]. Conversely, the C/N ratio follows opposite pattern to the SOC content with the highest (12.62) under ACZ 3 and the lowest (8.66) under ACZ 1.

The content of AP in the soil ranges from as low as 2.82 mg/kg in ACZ 3 to as high as 20.20 mg/kg in ACZ 1 showing highly significant variation (*p* < 0.01) across agro-climatic zones. Overall, the AP content of the soil is rated as low under ACZ 3 and high under ACZ 1 and ACZ 2. The AP content somehow reflects variations in the soil pH. AP is lowest in the agro-climatic zones with lowest pH suggesting the problem of P-fixation in the strongly acidic reactions. Conversely, the CEC ranges from 20.75 cmol/kg in the soils under ACZ 3 to 28.67 cmol/kg in the soils under ACZ 1. The mean CEC of the soil under ACZ 1 (28.67 ± 2.72) was significantly higher compared to the mean CEC of the soil under ACZ 3 (20.75 ± 1.32). However, the mean CEC of the soil under ACZ 2 was not significantly varied with the mean CEC under ACZ 1 and ACZ 3. Overall, the CEC of the soil is rated as medium under ACZ 2 and ACZ 3 but it is high under ACZ 1. This is a mirror image of the distribution of the SOC content (Table 3).

### Habitat gradient effect on soil physical and chemical properties

The edge and interior habitat were significantly (*p* < 0.05) affected some by soil physical and chemical properties at (Table 4). The mean values of sand (55.43 ± 2.48), silt (25.39 ± 2.31) and clay contents (19.78 ± 1.72) as well as the Si/Cl ratio (1.28 ± 0.07) in the soils of edge habitat were not significantly different compared to the mean sand (54.52 ± 2.44), silt (24.79 ± 2.30) and clay (20.24 ± 1.65) contents as well as Si/Cl ratio (1.22 ± 0.04) in the soils of the interior habitat. However, the mean BD of the soil in the edge habitat (0.75 ± 0.07) was significantly higher compared to the soils in the interior habitat (0.64 ± 0.02). Conversely, the contents of TN (0.56 ± 0.05), AP (9.82 ± 1.69) and C/N ratio (11.42 ± 0.84) in the edge habitat were significantly different compared to the contents of TN (0.73 ± 0.06), AP (12.81 ± 1.33) and C/N ratio (9.08 ± 0.23) in the soils of interior habitat. However, the mean pH, SOC, and CEC of the soil in the edge and interior habitat didn’t show significant different. Whereas, the mean pH (5.62 ± 0.16), CEC (23.67 ± 2.18) and SOC (6.63 ± 0.25) were markedly higher in the interior than the mean pH (5.58 ± 0.15), CEC (20.19 ± 1.23) and SOC (6.40 ± 0.22) in the edge habitat.

**Table 4:** The mean (± SE) values of selected soil physical properties as affected by habitat gradients

Soil properties	Habitat gradients		Mean	*F*	Sig
	Edge habitat	Interior habitat			
Sand (%)	55.43 ± 1.04	54.52 ± 0.99	54.97 ± 0.71	1.45	ns
Silt (%)	25.39 ± 1.34	24.79 ± 1.20	25.09 ± 0.89	1.04	ns
Clay (%)	19.18 ± 1.97	20.69 ± 1.57	19.94 ± 1.25	0.76	ns
Si/Cl	2.31 ± 0.57	1.44 ± 0.15	1.88 ± 0.30	0.082	ns
BD (g/cm.^3^)	0.75 ± 0.08^a^	0.64 ± 0.06^b^	0.69 ± 0.05	8.09	*
pH (H_2_0)	5.58 ± 0.11	5.62 ± 0.11	5.60 ± 0.08	1.04	ns
SOC (%)	6.40 ± 0.38	6.63 ± 0.39	6.51 ± 0.27	0.57	ns
TN (%)	0.56 ± 0.08^b^	0.73 ± 0.08^a^	0.64 ± 0.06	15.90	*
C/N ratio	14.65 ± 1.59^a^	10.67 ± 0.74^b^	12.65 ± 0.91	6.35	*
AP (mg/kg)	9.82 ± 1.05^b^	12.81 ± 1.59^a^	11.31 ± 0.97	14.69	**
CEC (cmol/kg)	20.19 ± 1.49	23.67 ± 1.62	21.93 ± 1.12	0.76	ns

Means in each column with the same superscript letter are not significantly different at *P* ≤ 0*.*05; *BD*  bulk density, *SOC*  organic carbon, *TN*  total nitrogen, *C/N*  carbon to nitrogen ratio, *AP* available phosphorus, *CEC* cation exchange capacity, *ns* nonsignificant; ^∗∗^*p* < 0*.*01 and ^∗^*p* < 0*.*05

## Discussion

### Soil physical and chemical properties across the natural vegetation

The soil properties are changing in an area due to the dynamic interactions between microclimatic conditions, vegetation types, and altitude [27, 28]. This follows from the pedogenetic factors that dictate soil formation including topographic factors, climate, biota along with parent materials operating over time as elaborated by Jenny [29]. The soil textural differences across the vegetation types might be due to the natural variations in the rate of weathering and some micro-topographical differences such as percentage slope instead of land management practices [30, 31].

The sand content of the soil in ericaceous land was significantly (*p* < 0.05) higher, compared to other vegetation types which might be due to the higher slope gradient (> 30%) under this land cover type that results in the erosional removal of fine particles down the slope. Consequently, the top soil that constitutes fine soil particles, such as silt and clay, could easily be eroded by the rain and leads for the high proportion of sand content in the area overtime. This is consistent with the high clay content in the moist forest that is located in the lower slope gradient positions. The highest value of clay in moist forest could be due to the high conversion rate of litter in the soil and the lowest value of clay content in the ericaceous land could be due to lower conversion rate of litter in the soil. However, the overall average clay content of the BMNP was less than 20%. Across all land cover type the soil BD was higher in the moist forest and lower in the ericaceous land. This result was consistent with the report by Yimer [32] that described ericaceous vegetation constituted the least BD compared to other vegetation types.

The lower level of pH in the grassland and afro-alpine land might be due to the high amount of rainfall and low level of temperature and that resulted for high moisture content in the soil. These areas are waterlogged and their watershed is characterized by flat, swampy areas, and many small shallow lakes, that are crucial for stream and river flow regulation into the lowlands, are situated [33]. The mean annual rainfall in these areas ranged from 1000 to 1400 mm and the mean annual minimum and maximum temperatures are 2.4 °C and 15.5 °C, respectively [33, 34]. The higher water holding capacity of the soil in those areas make cations easily solubilized and contributed for high pH level. Overall, the soils investigated in this study were characterized as strongly acid to moderately acid and its pH was ranged from 5.21–5.97 [26].

The SOC content showed significant variation with the change in vegetation types. The significantly higher content of SOC in ericaceous land, woodland and moist forest might be related to the higher decomposition rate of organic matter under higher temperatures and increased vegetation abundance that produced lots of leaf litter available for decomposition under this land cover type [32]. Recurrent fire in the ericaceous land could be the other reason for the higher amount of SOC in this land cover type since the burning of above ground biomass can add more carbon content into the soil [35]. Conversely, the reduced amount of SOC in the afro-alpine vegetation could be due to its lower temperature owing to its location in the higher altitude and the resulted lower decomposition rate of organic matters by the reduced bacterial activities [36]. Moreover, this vegetation type is covered with small size plants, mainly herbs and shrubs; as a result, less amount of organic matter is produced and added into the soil.

The strong and positive correlation (r = 0.75, *p* < 0.05) between the amount of TN and SOC in the BMNP strengthen the fact that most nitrogen forms are a part of the soil organic matter [37]. A C/N ratio above 12–14 is often considered indicative of a shortage of nitrogen in the soil [38]. However, this ratio in the study area was below the range and it indicates the high amount of nitrogen in the soil of the study area. Conversely, the amount of AP was varied significantly across the vegetation type and it was higher in the woodland, ericaceous land, moist forest and coffee forest which are located in the lower and middle altitudinal ranges, compared to Afro-alpine and grassland, which are located in the higher altitude. The significant variation in the amount of AP across the vegetation types at different altitudinal ranges could be due to the geology of the area and the nature of the soil [39, 40]. Weinert and Mazurek [41] suggested that the higher content of phosphorus in the soil could be related to the faster mineralization and mobilization of phosphorus. The downslope leaching from the higher altitudes could also be the other reason for the higher content of AP in the lower altitude vegetation types [32, 42]. The significantly lower level of AP in the grassland and Afro-alpine vegetation could be due to the problem of P-fixation as a result of lower pH level as well as due to the lower rate of organic matter decomposition. When the soil acidity increases, phosphorus availability decreases as it got fixed by the iron and aluminum oxides that are high in the soil solutions with strongly acidic reaction [40]. Soils with high clay content may have the ability to neutralize the acid-extracting solution and thus reduce the amounts of extractable phosphorus [43]. Phosphorus fixation tends to be higher and ease of phosphorus release tends to be lower in soils with higher clay contents [44].

The CEC of the soil in the study area was significantly higher in those vegetation types shown high level of SOC, such as ericaceous land, moist forest and woodland. Certain soil minerals, particularly clay in combination with organic matter, determine the soil CEC by attracting and holding oppositely charged ions [45]. Similar research reports were also made by Tegene [46], Eshetu et al., [47] and Yimer, [32] in the Ethiopian highlands. The higher level of soil pH in the ericaceous land, moist and coffee forest could be drawn from the relatively high amount of SOC and CEC as well as the high rate of organic matter decomposition in this vegetation types. Moreover, both moist and coffee forest are situated in the lower altitude and received relatively lower amount of rainfall (from 600–1000 mm) and had higher mean annual maximum temperature (29.1 °C) compared to the middle and higher altitudes [33, 34].

### Effects of land use/land cover change on soil physical and chemical properties

Human induced land cover change and habitat fragmentation owing to the expansion of farmlands and settlements significantly affected most of the soil physical and chemical properties in the BMNP. The clay content in the natural vegetation was significantly (*p* < 0.05) higher compared to the farmlands. The soil BD in the native vegetation, mainly in the moist and coffee forests, was higher, but not significant (*p* < 0.05), compared to the farmland and this could be due to the effect of high soil organic matter accumulation in the natural vegetation. However, the soil BD was higher in the farmland compared to the grassland, wood land, afro-alpine and ericaceous land. Soil compaction due to farming and livestock grazing could be the reason for the higher BD [48]. Trampling by cattle has been identified as the primary cause of high BD by compacting the soil surface [49].

The conversion of natural vegetation into farmland tends to decrease the soil pH [50]. In this study, the decrease in the soil pH was more pronounced in the farmland. The strongly acidic soil reaction under farmland is partly explained by the application of acid forming fertilizers such as DAP and urea for cereal cultivation. Moreover, the SOC and TN in the soils of the farmland were significantly (*p* < 0.05) lower compared to the natural vegetation. This might be due to the lower amount of organic material returned into the soil system, reduced litter decomposition rates, and high rates of soil organic matter oxidation due to tillage in the farmland [51, 52]. The higher amounts of SOC and TN in the natural vegetation were due to the higher accumulation of organic matter, which were resulted from the increased above and below-ground biomass [53, 54].

Conversely, land use changes significantly affected the CEC of the soil in the BMNP. The CEC of the farmland soil were found lower by 40% than the natural forest. Reports from different study revealed 27 to 43% reduction in soil CEC of the agricultural land compared with the natural vegetation [32, 54]. Thus, it is ascertained that the soil quality deterioration in the BMNP was mainly due to the human induced LULCC.

### The influence of altitudinal variation on soil physical and chemical properties

One of the leading factors governing soil organic matter accumulation and turnover is the mean annual temperature and precipitation [55, 56]. The mean annual precipitation of the BMNP area varied from 450 to 2400 mm. The amount of temperature in an area is affected by altitude. As a result, temperature decreases with increasing altitude and vice versa. Accordingly, lower decomposition rate has been asserted at higher altitude above 2800 m asl, i.e., ACZ 3, and higher decomposition rate has been recognized at lower altitude from 1600 to 3000 m asl, i.e., ACZ 1, in the study area. Higher temperature facilitates the decomposition rate of soil organic matter while lower temperature retards it. Large size trees were dominant in the ACZ 1 and that resulted for the high litter production. Whereas, herbaceous and shrub species were dominant in the ACZ 3 and that resulted for the lower litter production. Accordingly, the amount of SOC decreases as altitude increases and vice versa. Based on the FAO [39] assessment, the higher elevation plains of the Bale mountains are relatively infertile well drained Eutric Cambisols, whereas on the gently sloping foothills, below the escarpment, are relatively fertile Eutric nitisols.

In the same vein soil properties showed strong and positive correlation among themselves including soil pH and AP (r = 0.71, *p* < 0.01); SOC and TN (r = 0.75, *p* < 0.01); SOC and CEC (r = 0.74, *p* < 0.01); and TN and CEC (r = 0.67, *p* < 0.05). When the soil acidity increases, phosphorus availability decreases as it got fixed by iron and aluminum oxides and hydroxides that are high in the soil solutions with strongly acidic reaction [40]. As would be expected, the contents of TN are the mirror image of SOC that depending on the decomposition and mineralization of soil organic matter as evidenced by the high correlation coefficient [32, 40]. The positive and strong correlation between CEC and SOC is expected because soil organic matter and humus are one of the major sources of charge sites on the clay lattice in addition to the silicate mineral sources of negative charge sites that contribute towards the effective CEC. Although not significant, SOC is positively correlated with pH suggesting that the radical groups (e.g., –COO^−^) fix the H^+^ in the soil solution and increase the pH which is the negative logarism of the H^+^ concentration in the soil solution [40].

### Habitat gradient effect on soil physical and chemical properties

Some of the soil physical and chemical properties in the BMNP were significantly (*p* < 0.05) affected by the edge habitat. Accordingly, the soil sand and silt content as well as BD were higher in the edge habitat, whereas, the amount of the soil chemical properties assessed, including total N, SOC, CEC and AP were higher in the interior habitat. This result was similar with the report made by Zhou et al., [49] and Ruwanza [57]. This could be due to the exploitation and conversion of vegetation in the edge habitat through grazing, agriculture, and settlement expansion. The soils in the edge habitat were more compacted and less fertile. This could be due to the human activities, such as trampling by humans and livestock, agricultural activities and settlement expansion, in the edge habitat. As a result, vegetation in the edge habitats are destructed and less amount of litter content are added. Conversely, the soils in the interior 13habitats were porous and more fertile [58, 59]. This could be due to the less human disturbance and high accumulation of litter content in the interior habitat.

## Conclusion

Most of the soil physical and chemical properties variation in the BMNP is associated with the change in landscape structure, vegetation and habitat gradient. It was also affected by the change in temperature and precipitation due to altitudinal variation. Land use change were the main anthropogenic factors responsible for the change in soil properties in the study area. However, the soils of natural vegetation are characterized as fertile and rich in minerals (particularly ericaceous land, woodland and moist forest) compared with the farmland due to their high levels of pH, SOC, TN, AP and CEC. This was due to the return of high amount of litter from the aboveground biomass into the soil system through decomposition. It also attained low bulk density, which can potentially lead to high porosity and moisture-holding capacities that. This situation creates good aeration in the soil and enable plants to develop deep rooting system that assistance the plant root to penetrate the soil deeply and anchored in the ground firmly. Conversely, higher altitudes in the study area were observed to have higher amount of SOC and TN compared with lower altitudes, suggesting that these properties were regulated mainly by the mean annual temperature. Moreover, most of the soil physical and chemical properties were significantly affected by the edge. Accordingly, the amount of most of the soil physical properties was higher in the edge habitat, whereas, the amount of many of the soil chemical properties such as SOC, TN, AP and CEC were higher in the interior habitat. The soils in the edge habitat were more compacted and less fertile whereas the soils in the interior habitats were porous and more fertile. This was due to the exploitation and conversion of vegetation in the edge habitat through grazing, agriculture, and settlement expansion.

The vegetation and soils of the park should be properly managed and protected so as to withstand the effects of climate change due to the release of CO_2_ from deforestation and forest degradation. Conservation and restoration priority should be given to those vegetation types and ecosystems that are highly affected by human interferences such as the grassland in the middle altitude, ericaceous land in the higher altitude, and moist forest in the lower altitudes for better ecosystem management practices and thereby improving the potential of the physical and biological resources in the study area. The increasing trends of edge habitat should be retarded by limiting the natural resource use and the human activities in the park area to improve the vegetation composition, structure, and soil properties. Degraded habitats need to be restored by avoiding human contact and excessive burden on natural resources of the park.

## Data Availability

The datasets used and/or analyzed during the current study available from the corresponding author on reasonable request.

## Abbreviations

ACZ: Agro-climatic zones
AP: Available phosphorous
BD: Bulk density
BMNP: Bale Mountains national park
CEC: Cation exchange capacity
GLM: Generalized linear model
HSD: Tukey’s honest significance difference
HG: Habitat gradient
LSD: Least significant difference
LCT: Land cover type
SOC: Soil organic carbon
TN: Total nitrogen
